# Metal-Exchanged β Zeolites as Catalysts for the Conversion of Acetone to Hydrocarbons

**DOI:** 10.3390/ma5010121

**Published:** 2012-01-05

**Authors:** Aurora J. Cruz-Cabeza, Dolores Esquivel, César Jiménez-Sanchidrián, Francisco J. Romero-Salguero

**Affiliations:** Departamento de Química Orgánica, Instituto Universitario de Investigación en Química Fina y Nanoquímica (IUIQFN), Universidad de Córdoba, Campus de Rabanales, Edificio Marie Curie, Ctra. Nnal. IV, km 396, Córdoba 14014, Spain; E-Mails: aurorajosecruz@gmail.com (A.J.C.-C.); doloesquivel@hotmail.com (D.E.); qo1jisac@uco.es (C.J.-S.)

**Keywords:** zeolite β, ion exchange, characterization, acidity, pyridine, acetonitrile, acetone conversion, hydrocarbons, isobutene, mesitylene

## Abstract

Various metal-β zeolites have been synthesized under similar ion-exchange conditions. During the exchange process, the nature and acid strength of the used cations modified the composition and textural properties as well as the Brönsted and Lewis acidity of the final materials. Zeolites exchanged with divalent cations showed a clear decrease of their surface Brönsted acidity and an increase of their Lewis acidity. All materials were active as catalysts for the transformation of acetone into hydrocarbons. Although the protonic zeolite was the most active in the acetone conversion (96.8% conversion), the metal-exchanged zeolites showed varied selectivities towards different products of the reaction. In particular, we found the Cu-β to have a considerable selectivity towards the production of isobutene from acetone (over 31% yield compared to 7.5% of the protonic zeolite). We propose different reactions mechanisms in order to explain the final product distributions.

## 1. Introduction

As a result of increasing oil prices, the conversion of different low molecular weight organic molecules into gasoline has attracted much attention in recent years [1,2]. Among those precursors of gasoline, different C_1_-C_4_ oxygenates are good candidates. In particular, acetone constitutes a real alternative source of hydrocarbons since acetone is readily available as a secondary product in propylene oxide production and in phenol synthesis from cumene [3]. There is currently a critical need for the development of commodity chemicals, energy, and materials from renewable biobased feedstocks. Acetone can be produced from abundantly available biomass, such as agricultural wastes, by ABE (acetone-butanol-ethanol) fermentation [4]. During the first part of the 20th century the anaerobic production of ABE by solventogenic clostridia, aimed at the production of acetone for the war industry, was the second largest biotechnological process in the world. Although the petroleum-based production of solvents replaced this process, the shortage of fossil fuels in the near future has revived interest in the subject and much effort is now being devoted to improve ABE production from biomass [5]. The catalytic steam reforming of bioethanol also produces acetone and hydrogen by using catalysts such as CuO/CeO_2_ [6]. Acetone is also an important oxygenate component of the biomass pyrolysis oils, whose up-grading by reduction of their oxygen content is actively pursued [7,8]. Very recently, Tago *et al.* [9,10,11] have reported the chemical transformation of different biomass wastes, such as sewage sludge, fermentation residues and livestock manure, into acetone by using a ZrO_2_-FeO_x_ catalyst.

The transformation of acetone into hydrocarbons is a complex process which seems to takes place via acetone aldolization and dehydration followed by cyclization, aromatization and cracking, among other reactions [12,13]. Acetone has been converted into gasoline through a great variety of heterogeneous acid and basic catalysts [14] including several acid and basic zeolites [15,16]. Hutchings *et al.* [17] compared the catalytic activity of the proton forms of zeolites β and ZSM-5 in the transformation of acetone into hydrocarbons. They demonstrated that high isobutene selectivities can be achieved at high acetone conversion using zeolite β as catalyst, in contrast to the low selectivity attained with zeolite ZSM-5. Although they reported a selectivity to C6+ below 3% for an acetone conversion close to 65%, other authors in later studies found much higher conversion of acetone to aromatics (over 30% in some cases) [18,19]. Very recently, the conversion of acetone to hydrocarbons has also been investigated on alkaline and alkaline-earth exchanged beta zeolites [18,19]. The exchanged samples exhibited a higher selectivity to isobutene than the protonic zeolite, in particular those containing potassium and barium as metal cations.

Many catalytic applications of zeolites involve the modification of their acid-base and redox properties by exchange with different metal cations [20]. Although extensively investigated for many zeolitic structures, metal cation-exchange on zeolite β has been studied less [21]. Herein we describe the ion exchange of zeolite β with different metal cations and their use as catalysts in the transformation of acetone into hydrocarbons. The materials have been characterized and the aliphatic and aromatic compositions of the products in the catalyzed reaction have been determined in order to evaluate the influence of the exchanged metals in the activity and selectivity of zeolite β.

## 2. Results and Discussion

### 2.1. Characterization

**Figure 1:**
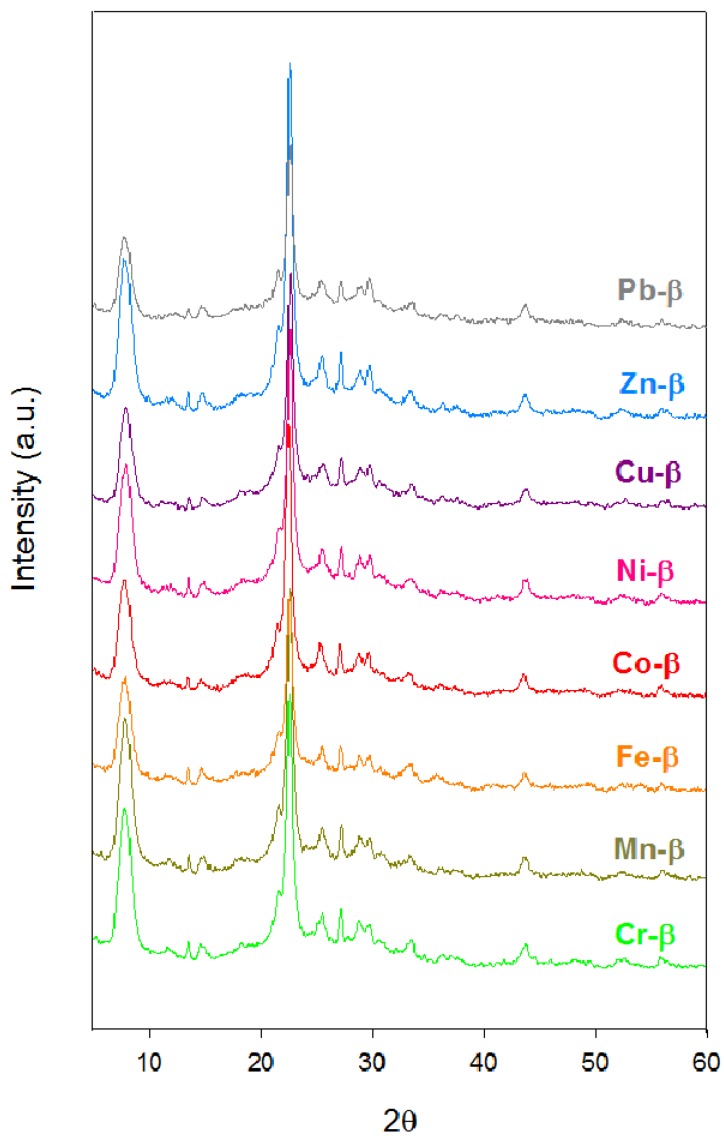
Powder X-ray diffraction patterns for metal exchanged β zeolites.

The XRD patterns of the metal exchanged zeolite β (Figure 1) were almost identical to that of the protonic zeolite [22]. No diffraction lines corresponding to oxide crystallites were observed, thus indicating that metal cations were well dispersed on the zeolite. However, there was a certain loss of crystallinity (Table 1), which was particularly significant for the Fe-β and Pb-β materials. Silicon to aluminium ratios slightly increased for most exchanged zeolites compared to that of the parent zeolite β (Table 1). Two of the new materials, however, underwent considerable dealumination, *i.e.*, the chromium and the iron exchanged samples. The pK_a_ values for Cr^3+^ and Fe^3+^ are 4.0 and 2.2 respectively, whereas they range between 9 and 11 for the remaining cations. Clearly, the Si/Al ratio increased with the acidic character of the exchanged cations.

Even though the same exchange procedure was used for all samples, each metal cation was incorporated to zeolite β in a different extension. Cu^2+^ and Pb^2+^ were almost quantitatively exchanged giving rise to very high exchange degrees (98% and 84%, respectively). The lowest metal to aluminum ratios were obtained after exchange with Mn^2+^ and Ni^2+^, whereas Zn^2+^ and Co^2+^ also had a significant Me/Al ratio. The high content in Fe^3+^ (4.8% of the total sample weight) revealed the presence of such metal not only as an exchanged cation but also as oxide-like species consisting of very small (<3–4 nm) or disordered crystallites [23].

**Table 1:** Some characteristics of metal exchanged β zeolites ^1^.

Catalyst	Si/Al ratio	Metal content (%)	Me/Al ratio	Exchange degree (%)	Crystallinity (%)
H-β	12.5	-	-	-	100
Cr-β	27.9	1.00	0.44	-	94
Mn-β	15.3	0.89	0.23	46	83
Fe-β	49.9	4.80	3.77	-	64
Co-β	13.7	1.44	0.35	70	90
Ni-β	15.5	0.80	0.21	43	81
Cu-β	13.7	2.06	0.48	98	84
Zn-β	16.2	1.22	0.32	64	99
Al-β	14.5	-	-	-	86
Pb-β	16.7	1.50	0.42	84	65

^1^ See experimental section for details.

**Figure 2:**
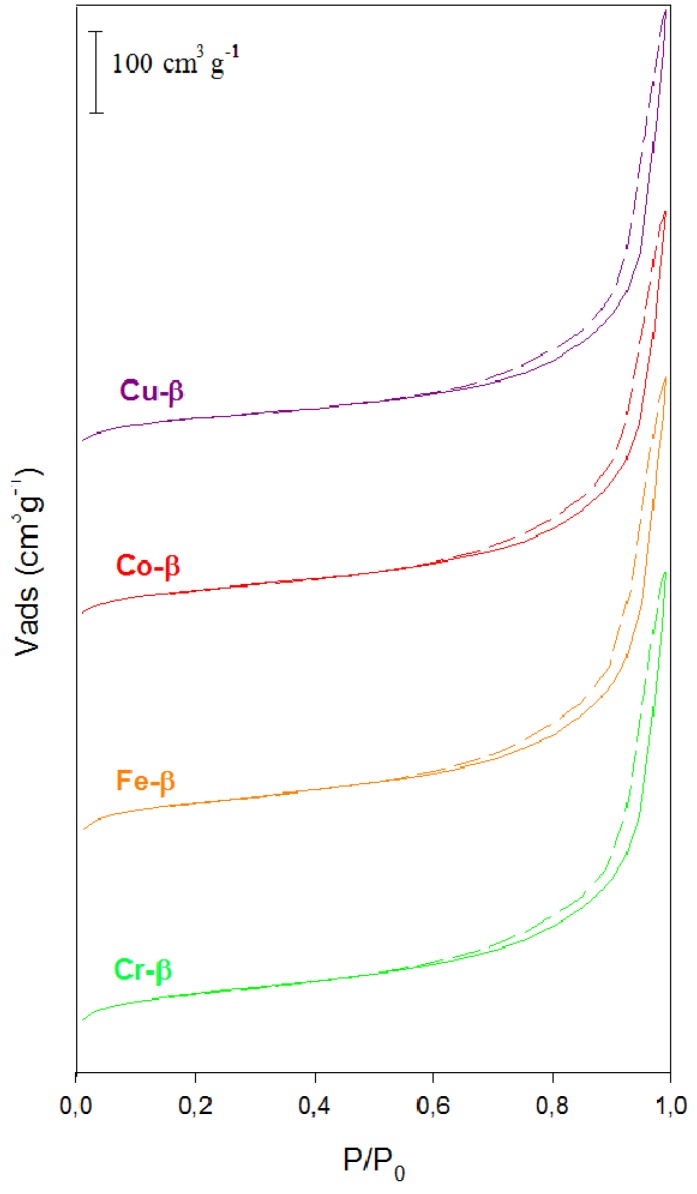
Representative N_2_ adsorption-desorption isotherms for metal exchanged β zeolites.

All zeolites exhibited a combined type I and IV isotherm adsorption behavior due to the presence of zeolitic micropores as well as mesopores formed by the aggregation of crystals (Figure 2). No new mesoporosity was generated after the ion exchange treatment. All samples experienced a non-negligible decrease in their surface area (Table 2). In general, both micropore and mesopore volumes were smaller for the exchanged zeolites than for sample H-β. However, this decrease in volume was more pronounced for the micropores (*ca*. 20% compared to 10% as much for mesopores) as a result of the preferential location of the exchanged cations inside the micropores of the zeolite.

**Table 2:** Surface properties of metal exchanged β zeolites.

Catalyst	S_BET_ (m^2^ g^−1^)	Mesopore volume (cm^3^ g^−1^)	Micropore volume (cm^3^ g^−1^)
H-β	582	0.89	0.22
Cr-β	540	0.88	0.19
Mn-β	531	0.90	0.19
Fe-β	513	0.89	0.18
Co-β	513	0.79	0.19
Ni-β	505	0.84	0.17
Cu-β	499	0.84	0.18
Zn-β	525	0.89	0.19
Al-β	522	0.85	0.19
Pb-β	545	0.85	0.18

**Figure 3:**
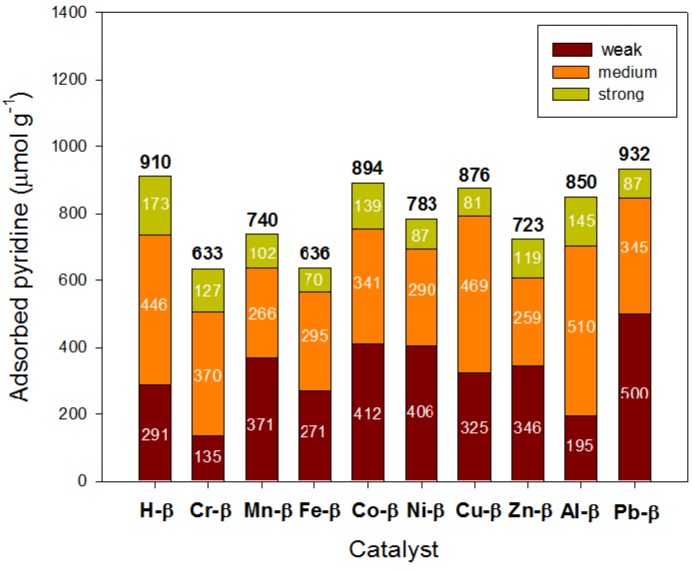
Surface acidity of metal exchanged β zeolites determined by thermal programmed desorption of pyridine.

Figure 3 depicts the population of acid sites determined by pyridine desorption. All materials adsorbed a significant amount of pyridine. Pyridine can be adsorbed in both Brönsted and Lewis acid sites [19]. Zeolites Cr-β and Fe-β showed the lowest pyridine adsorption due to their higher Si/Al ratios caused by their dealumination during ion-exchange. The zeolites with the greatest number of strong and medium acid sites were H-β and Al-β whereas metal exchanged zeolites showed higher populations of weak acid sites. Thus, those weak Lewis acid sites generated after ion exchange desorbed pyridine at lower temperatures. Zeolites exchanged with trivalent cations (Cr^3+^, Fe^3+^ and Al^3+^), however, did not exhibit such an increase in the population of weak acid sites whilst the Cu-β sample, which was almost quantitatively exchanged, had Lewis sites able to adsorb pyridine up to 350–550 °C.

**Figure 4:**
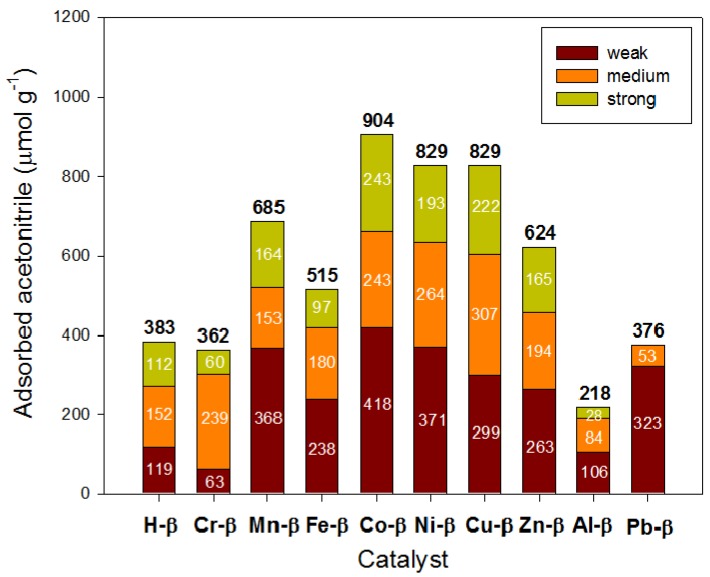
Surface acidity of metal exchanged β zeolites determined by thermal programmed desorption of acetonitrile.

The Lewis acid character of the exchanged metal cations was first revealed by acetonitrile adsorption studies (acetonitrile is preferentially adsorbed on Lewis acid sites) [24,25]. In the protonic zeolite (H-β), aluminum defects are the only sites responsible for Lewis acidity. Through the incorporation of metal cations, to the exception of the Cr^3+^ and particularly Al^3+^ samples, the increase in the number of Lewis acid sites and, therefore, the amount of adsorbed acetonitrile was remarkable in most cases (Figure 4). The highest populations of Lewis sites were found for the samples exchanged with Co^2+^, Ni^2+^ and Cu^2+^. The marked decrease in Lewis acidity for sample Al-β has been recently explained by the reduction of the octahedrally coordinated aluminium atoms [22]. Also, strong sites were absent in zeolite Pb-β, similar to alkaline-earth exchanged zeolite β [19].

Overall, the acidity measurements indicated that cations were preferentially exchanged in medium and strong Brönsted acid sites of zeolite β, thus generating new Lewis acid sites. Divalent cations gave rise to stronger Lewis sites than trivalent cations probably because the latter (specifically, Cr-β and Fe-β) were not really exchanged but mainly present as oxide-like clusters.

### 2.2. Catalytic Activity

All metal exchanged β zeolites were active in the transformation of acetone into hydrocarbons (Table 3) but none of them showed higher activity than the protonic one. Specifically, the catalytic activity decreased in the following order: H-β > Al-β > Mn-β, Cr-β, Zn-β > Cu-β > Pb-β, Ni-β, Co-β, Fe-β. Although the most active catalysts, *i.e.*, H-β and Al-β, were those exhibiting the largest population of strong and medium Brönsted acid sites, we found no clear relationship between acidity and the acetone conversion or its selectivity. Two reasons could explain this observation: on one hand, numerous reactions can be involved in this process; on the other hand, both Brönsted and Lewis acid sites can participate in activating acetone [15,19]. Certainly, Lewis acid sites influenced the reaction because the acetone conversion was not proportional to the population of Brönsted acid sites, in agreement with previous observations by Flego *et al.* [26] for a variety of molecular sieves with different acidity and porosity.

**Table 3:** Catalytic activity of metal exchanged β zeolites in the conversion of acetone at 400 °C.

Catalyst	Conversion (%)	Relative yields (wt %)			
		CO_2_	Aliphatics ^1^	Aromatics	
				Benzene derivatives ^2^	Naphthalene derivatives ^3^
H-β	96.8	6.7	37.6	40.4	12.1
Cr-β	81.5	5.6	32.5	36.4	7.0
Mn-β	81.9	6.0	35.7	40.2	-
Fe-β	37.5	2.6	17.1	17.8	-
Co-β	39.6	3.3	28.3	8.0	-
Ni-β	42.0	3.7	29.6	8.7	-
Cu-β	58.5	16.6	41.9	-	-
Zn-β	79.5	4.3	29.0	42.7	3.5
Al-β	91.6	2.8	24.2	50.0	14.5
Pb-β	45.1	6.0	25.3	13.9	-

^1^ See Table 4 for a detailed analysis; ^2^ See Table 5 for a detailed analysis; ^3^ Fraction composed of naphthalene, methylnaphthalenes, dimethylnaphthalenes and trimethylnaphthalenes.

Under the current experimental conditions, the zeolite catalyzed acetone conversion yielded numerous reaction products but no oxygenated organic compounds were observed. The resulting hydrocarbons were classified into aliphatic and aromatic compounds in Table 3 whilst detailed compositions of both fractions are given in Table 4 and Table 5, respectively. The overall product composition highlights the complexity of the reactions involved in the acetone transformation. Scheme I shows some possible reaction pathways. The predominant presence of isobutene and trimethylbenzenes in the aliphatic and aromatic fractions, respectively, suggested that an acid-catalyzed aldol condensation is the primary reaction. Thus, acetone dimerization can give rise to diacetone alcohol that would yield isobutene and acetic acid after cracking. The latter can undergo further reactions, such as its cleavage into methane and carbon dioxide [16]. However, the formation of methane in all cases was negligible and so acetic acid would be likely to proceed via dehydration to give an acylium ion which later would undergo a nucleophilic attack by another molecule, thus resulting in acetone and CO_2_ [1]. This reaction may also occur via the reaction between acetic acid and ketene, which is known to be produced by decomposition of acetic acid in zeolites [27]. Moreover, 1,3,5-trimethylbenzene (mesitylene) could be formed by reaction of mesityl oxide and acetone on strong acid sites [28]. The presence of different benzene derivatives with up to 11 carbon atoms could be explained by assuming that mesitylene and the resulting aromatic compounds undergo several reactions, such as isomerizations, disproportionations and transalkylations [29]. Moreover, naphthalene derivatives could be obtained via oligomerization of acetone in a similar way. In addition, the presence of propene (main C3-^=^ compound) and C_4_ and C_5_ alkanes and alkenes could be explained by oligomerization, cracking and hydrogen transfer reactions. Ethylene was only detected in trace amounts.

**Table 4:** Relative yields (wt %) to the compounds present in the aliphatic fraction.

Catalyst	C3-^=^	iC4	iC4^=^	nC4	nC4^=^	C5-C6	C5-C6^=^
H-β	6.9	11.3	7.5	1.0	4.3	3.6	3.0
Cr-β	5.6	6.9	17.0	-	-	-	3.0
Mn-β	5.0	6.9	14.3	-	5.7	-	3.8
Fe-β	2.6	3.3	11.2	-	-	-	-
Co-β	1.5	1.2	23.2	-	1.3	-	1.1
Ni-β	6.8	5.1	16.0	-	1.7	-	-
Cu-β	7.5	2.1	31.3	-	-	-	1.0
Zn-β	4.2	4.7	14.6	-	1.7	-	3.8
Al-β	2.7	7.2	6.9	0.5	3.1	1.8	1.9
Pb-β	3.3	3.0	13.8	-	3.4	-	1.8

C3-^=^, ethene and propene; iC4, isobutane; iC4^=^, isobutene; nC4, n-butane; nC4^=^, butenes; C5-C6, alkanes with five and six carbon atoms; C5-C6^=^, alkenes with five and six carbon atoms.

**Table 5:** Relative yields (wt %) to the compounds present in the aromatic fraction.

Catalyst	B	T	A8	TMB	A9	TeMB	A10–A11
H-β	1.4	8.7	13.9	8.8	4.2	1.2	2.1
Cr-β	0.9	5.9	10.1	10.7	3.0	3.7	3.0
Mn-β	0.7	4.6	7.4	9.6	2.5	5.4	10.0
Fe-β	-	2.7	2.9	6.0	-	3.4	2.8
Co-β	-	1.1	-	3.1	-	-	3.8
Ni-β	-	4.3	2.6	1.8	-	-	-
Cu-β	-	-	-	-	-	-	-
Zn-β	1.3	7.0	11.8	11.4	3.6	2.9	4.7
Al-β	1.2	7.9	14.9	14.9	3.8	2.6	4.6
Pb-β	-	3.4	3.9	6.6	-	-	-

B, benzene; T, toluene; A8, C8 benzene derivatives; TMB, trimethylbenzenes; A9, C9 benzene derivatives, except for TMB; TeMB, tetramethylbenzenes; A10–A11, C10 and C11 benzene derivatives, except for TeMB.

Zeolites H-β and Al-β yielded the widest range of products (Table 4 and Table 5). The Al-β sample gave the highest selectivity toward aromatics (ca. 70%) whereas Cu-β was virtually inactive for the production of these compounds. Together with Co-β and Ni-β, Cu-β was the most selective towards aliphatics (ca. 70%). Catalyzed by Cu-β, isobutene was obtained from acetone with a selectivity as high as 54%.

**Figure 5:**
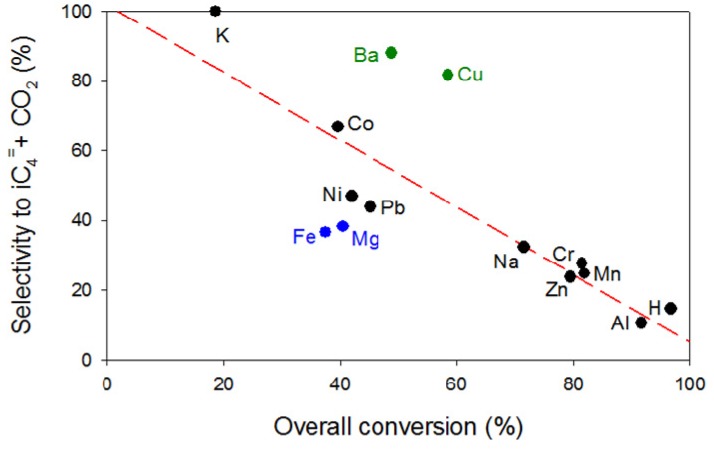
Relationship between the overall conversion and the selectivity to isobutene and CO_2_ in the transformation of acetone into hydrocarbons on metal exchanged zeolite β at 400 °C.

The selectivity to isobutene and CO_2_ (both coming directly from the diacetone alcohol cracking) was plotted against the overall conversion of acetone for each catalyst (Figure 5), together with some alkaline and alkaline-earth metal exchanged zeolites reported previously [19], for which a low fit linear relationship was found. Zeolites Mg-β and Fe-β behaved as outliers from this linear trend exhibiting lower selectivities than expected due, perhaps, to the presence of metal oxide-like structures as discussed above. In contrast, zeolites Ba-β and Cu-β yielded very high selectivities to isobutene and CO_2_. Interestingly, Lewis acid sites of medium strength were prominent in both of these materials (Figure 4) [19] and strong Brönsted acid sites were only present in a small population, thus preventing isobutene from undergoing secondary reactions such as oligomerization, aromatization and cracking. Due to the outstanding importance of isobutene as an intermediate chemical, both these catalysts, Ba-β and Cu-β, could be of a great commercial use [18,30]. The H-β and Al-β zeolites showed the highest isobutane to isobutene ratios among all catalysts due to their higher population of medium and strong Brönsted acid sites able to catalyze the disproportionation of alkenes to alkanes and arenes [31]. Additionally, alkene oligomerization is catalyzed by weak–medium acid sites, whereas alkylation, hydrogen transfer and cracking reactions are promoted by strong acid sites [32], all these reactions being responsible for the formation of aliphatic compounds.

**Scheme I:**
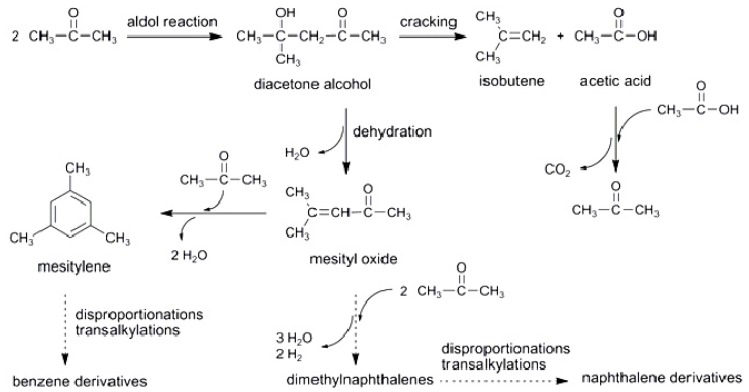
Some of the possible reaction pathways in the acetone conversion to hydrocarbons.

**Figure 6:**
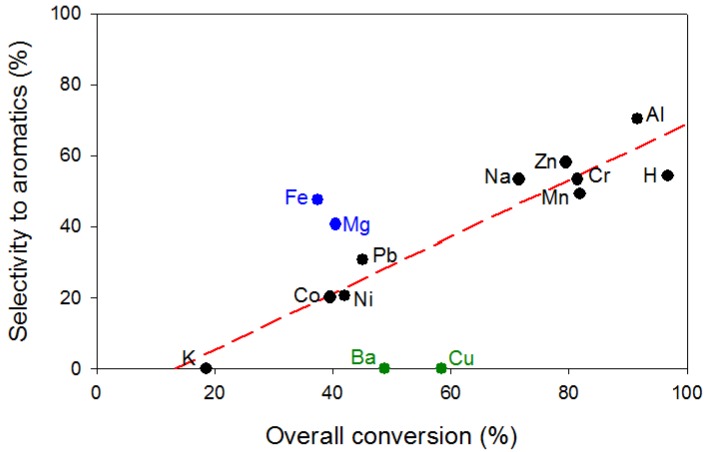
Relationship between the overall conversion and the selectivity to aromatic compounds in the transformation of acetone into hydrocarbons on metal exchanged zeolite β at 400 °C.

Figure 6 depicts the selectivity to aromatics as a function of the overall acetone conversion. Very active catalysts (with conversions > 70%) gave rise to high selectivities to aromatics between 50–70% whereas those with intermediate conversions (~40%) exhibited selectivities in the range of 20–30%. Mg-β and Fe-β as well as Ba-β and Cu-β were the exceptions to the trends due to the reasons mentioned above. The two latter produced no aromatic compounds, similarly to K-β which showed a much lower conversion.

Among the most active zeolites, the Mn-β sample was the only one to produce no naphthalene derivatives even though the Mn-β sample gave rise to a significant contribution of tetra and pentamethylbenzenes (30% of the aromatic fraction compare to a 3% found in most cases). Zeolites Al-β and H-β, as well as Cr-β and Zn-β to a less extent, yielded the higher amount of naphthalene derivatives (Table 3). Methylnaphthalenes and dimethylnaphthalenes were the most abundant compounds in this fraction (80–100%). Also, small amounts of naphthalene and trimethylnaphthalenes were detected.

Remarkably, shape-selectivity seemed to play an important role through steric restrictions in the pores since zeolites Ni-β and Pb-β, exhibiting the smallest micropore volumes, did not yield aromatic compounds with more than 9 carbon atoms which involve the formation of bulkier intermediates.

## 3. Experimental Section

### 3.1. Ion-Exchange Procedure

Metal cation-exchanged zeolites were prepared from NH_4_-β(12.5) purchased from Zeolyst Int. (ref. CP814E). The commercialized zeolite was calcined at 600 °C (at a rate of 1 °C/min) for 3 h to obtain its protonic form. The ion-exchange procedure was performed by stirring a suspension of the zeolite in a 0.3 M aqueous solution of the metal salt of interest [*i.e.*, Cr(NO_3_)_3_, Mn(NO_3_)_2_·4H_2_O, Fe(NO_3_)_3_·9H_2_O, Co(CH_3_COO)_2_·4H_2_O, Ni(NO_3_)_2_·6H_2_O, Cu(CH_3_COO)_2_·H_2_O, Zn(NO_3_)_2_·6H_2_O, Al(NO_3_)_3_·9H_2_O and Pb(NO_3_)_2_] at 80 °C during 24 h using, approximately, 6 mL of liquid per gram of solid. After filtration under vacuum and repeatedly washing until the filtrate was free of metal ions, the exchanged zeolites were calcined at 600 °C for 3 h. All solids were sifted through 200–250 mesh.

### 3.2. Characterization of Catalysts

X-Ray powder diffraction (XRD) patterns were recorded on a Siemens D-5000 powder diffractometer (Cu-Kα radiation). Crystallinity was calculated by comparison of the peak areas at 2θ = 22.5 of the exchanged zeolites with that of the acidic β zeolite. Elemental compositions were determined by energy dispersive X-ray analysis (EDAX) on a Jeol JSM-5400 instrument equipped with a Link ISI analyser and a Pentafet detector (Oxford, UK). Textural properties were analyzed from N_2_ adsorption isotherms at 77 K on a Micromeritics ASAP 2000. S_BET_ and BJH mesopore volumes were calculated from the N_2_ adsorption data at relative pressures 0.1 < P/P_0_ < 1, whereas micropore volumes were obtained by using the Dubinin-Astakhov method (n = 4) at relative pressures 0.000001 < P/P_0_ < 0.1.

The acidity of the exchanged zeolites was determined by chemisorbing pyridine (at 140 °C) and acetonitrile (at 110 °C) and then applying temperature programmed desorptions (TPDs) [19]. Acid sites desorbing pyridine below 350 °C were considered as weak acid sites, whereas those in the range from 350 to 550 °C and above 550 °C were assigned to medium and strong acid sites, respectively. For acetonitrile, medium acid sites desorbed between 350 and 500 °C.

### 3.3. Catalytic Activity

Catalytic reactions were carried out at 400 °C in a microanalytical pulse reactor reported elsewhere [19]. Ten milligrams of catalyst were held in the 4-mm-diameter reactor tube using small plugs of glass wool. Helium was used as carrier gas at a flow rate of 100 mL min^−1^ and a pulse size of 0.2 µL. The analyses were performed on a Petrocol 100 m × 0.25 mm ID capillary column and the identity of each reaction product was determined by mass spectrometry using a Hewlett Packard 5971A mass selective detector. Conversions and selectivities were calculated on a carbon basis. No variation in the product composition was observed after five consecutive pulses. Although the pulse reactor has been shown to be of limited usefulness with respect to mechanistic studies, pulse reactors provide a simple technology for the rapid screening of different catalysts [33] and minimize the effect of coke deposition [34]. Also, the configuration of the system avoids the condensation of heavy aromatic products in the transmission lines.

## 4. Conclusions

A zeolite β was subjected to ion exchange with different divalent and trivalent metal cations under identical conditions. The resulting materials underwent ion-exchange in different extensions and their Si/Al ratios slightly increased (from 12.5 to values ranging from 13 to 17) in all cases except for the Cr^3+^ and particularly Fe^3+^ ions which caused a significant dealumination during the process (Si/Al ratios of almost 30 and 50%, respectively). Upon exchange, both the surface area and the micropore volume of the zeolites were reduced by a maximum of ~15% and ~22%, respectively. The ion exchange procedure also resulted in a decrease of Brönsted acidity, particularly for sites of medium and high strength, while new Lewis acid sites were generated. All materials were active in the transformation of acetone into hydrocarbons but no clear relationships between activity and acidity could be established since both Brönsted and Lewis acid sites seemed to be able to activate acetone. The reaction product selectivity was found closely related to the catalyst activity and some shape-selectivity effects were observed.

## References

[1] Chang C.D., Silvestri A.J. (1977). The conversion of methanol and other O-compounds to hydrocarbons over zeolite catalysts. J. Catal..

[2] Mentzel U.V., Holm M.S. (2011). Utilization of biomass: Conversion of model compounds to hydrocarbons over zeolite H-ZSM-5. Appl. Catal. A.

[3] Knifton J.F., Dai P.S.E. (1999). Diisopropyl ether syntheses from crude acetone. Catal. Lett..

[4] Jesse T.W., Ezeji T.C., Qureshi N., Blaschek H.P. (2002). Production of butanol from starch-based waste packing peanuts and agricultural waste. J. Ind. Microbiol. Biotechnol..

[5] Claassen P.A.M., van Lier J.B., Lopez Contreras A.M., van Niel E.W.J., Sijtsma L., Stams A.J.M., de Vries S.S., Weusthuis R.A. (1999). Utilisation of biomass for the supply of energy carriers. Appl. Microbiol. Biotechnol..

[6] Nishiguchi T., Matsumoto T., Kanai H., Utani K., Matsumura Y., Shen W.-J., Imamura S. (2005). Catalytic steam reforming of ethanol to produce hydrogen and acetone. Appl. Catal. A.

[7] Gayubo A.G., Aguayo A.T., Atutxa A., Aguado R., Olazar M., Bilbao J. (2004). Transformation of oxygenate components of biomass pyrolysis oil on a HZSM-5 zeolite. II. Aldehydes, ketones, and acids. Ind. Eng. Chem. Res..

[8] Stefanidis S.D., Kalogiannis K.G., Iliopoulou E.F., Lappas A.A., Pilavachi P.A. (2011). *In-situ* upgrading of biomass pyrolysis vapors: Catalyst screening on a fixed bed reactor. Bioresour. Technol..

[9] Fumoto E., Mizutani Y., Tago T., Masuda T. (2006). Production of ketones from sewage sludge over zirconia-supporting iron oxide catalysts in a steam atmosphere. Appl. Catal. B.

[10] Funai S., Satoh Y., Satoh Y., Tajima K., Tago T., Masuda T. (2010). Development of a new conversion process consisting of hydrothermal treatment and catalytic reaction using ZrO_2_–FeO_X_ catalyst to convert fermentation residue into useful chemicals. Top. Catal..

[11] Funai S., Tago T., Masuda T. (2011). Selective production of ketones from biomass waste containing a large amount of water using an iron oxide catalyst. Catal. Today.

[12] Baigrie L.M., Cox R.A., Slebocka-Tilk H., Tencer M., Tidwell T.T. (1985). Acid-catalyzed enolization and aldol condensation of acetaldehyde. J. Am. Chem. Soc..

[13] Novakova J., Kubelkova L., Dolejsek Z. (1987). TPD/MS studies of the interaction of simple ketones with ZSM-5 zeolites. J. Mol. Catal..

[14] Salvapati G.S., Ramanamurty K.V., Janardanarao M. (1989). Selective catalytic self-condensation of acetone. J. Mol. Catal..

[15] Panov A.G., Fripiat J.J. (1998). Acetone condensation reaction on acid catalysts. J. Catal..

[16] Veloso C.O., Monteiro J.L.F., Sousa-Aguiar E.F. (1994). Aldol condensation of acetone over alkali cation exchanged zeolites. Stud. Surf. Sci. Catal..

[17] Hutchings G.J., Johnston P., Lee D.F., Williams C.D. (1993). Acetone conversion to isobutene in high selectivity using zeolite β catalyst. Catal. Lett..

[18] Tago T., Konno H., Ikeda S., Yamazaki S., Ninomiya W., Nakasaka Y., Masuda T. (2011). Selective production of isobutylene from acetone over alkali metal ion-exchanged BEA zeolites. Catal. Today.

[19] Esquivel D., Cruz-Cabeza A.J., Jiménez-Sanchidrián C., Romero-Salguero F.J. (2011). Local environment and acidity in alkaline and alkaline-earth exchanged β zeolite: Structural analysis and catalytic properties. Micropor. Mesopor. Mat..

[20] Weitkamp J. (2000). Zeolites and catalysis. Solid State Ionics.

[21] Armor J.N. (1998). Metal-exchanged zeolites as catalysts. Micropor. Mesopor. Mat..

[22] Esquivel D., Cruz-Cabeza A.J., Jiménez-Sanchidrián C., Romero-Salguero F.J. (2012). Enhanced concentration of medium strength brönsted acid sites in aluminium-modified β zeolite. Catal. Lett..

[23] Mauvezin M., Delahay G., Coq B., Kieger S., Jumas J.C., Olivier-Fourcade J. (2001). Identification of iron species in Fe-BEA: Influence of the exchange level. J. Phys. Chem. B.

[24] Penzien J., Muller T.E., Lercher J.A. (2001). Hydroamination of 6-aminohex-1-yne over zinc based homogeneous and zeolite catalysts. Micropor. Mesopor. Mat..

[25] Pelmenschikov A.G., van Santen R.A., Jänchen J., Meijer E. (1993). CD_3_CN as a probe of lewis and bronsted acidity of zeolites. J. Phys. Chem..

[26] Flego C., Perego C. (2000). Acetone condensation as a model reaction for the catalytic behavior of acidic molecular sieves: A UV-Vis study. Appl. Catal. A.

[27] Parker L.M., Bibby D.M., Miller I.J. (1991). Formation of ketenes by reaction of carboxylic acids over alkali metal-exchanged zeolites. J. Catal..

[28] Olah G.A., Ip W.M. (1988). Catalysis of solid superacids. 23. Selective condensation of acetone to mesitylene over superacidic nafion-H and comparison with acidic-basic tungsten(VI) oxide/alumina or zirconium(IV) oxide/alumina catalysts. New J. Chem..

[29] Roldán R., Romero F.J., Jiménez C., Borau V., Marinas J.M. (2004). transformation of mixtures of benzene and xylenes into toluene by transalkylation on zeolites. Appl. Catal. A.

[30] Tago T., Konno H., Sakamoto M., Nakasaka Y., Masuda T. (2011). Selective synthesis for light olefins from acetone over ZSM-5 zeolites with nano- and macro-crystal sizes. Appl. Catal. A.

[31] Mikkelsen O., Kolboe S. (1999). The conversion of methanol to hydrocarbons over zeolite H-beta. Micropor. Mesopor. Mat..

[32] Corma A., Martinez A., Arroyo P.A., Monteiro J.L.F., Sousa-Aguiar E.F. (1996). Isobutane/2-butene alkylation on zeolite beta: Influence of post-synthesis treatments. Appl. Catal. A.

[33] Hutchings G.J., Lee D.F., Lynch M. (1993). Methanol conversion to hydrocarbons over zeolite H-ZSM-5: Comments on the formation of C_4_ hydrocarbons at low reaction temperatures. Appl. Catal. A.

[34] Itoh H., Hattori T., Murakami Y. (1982). Product distribution in the conversion of methanol on partially ion-exchanged mordenites. Appl. Catal..

